# Editorial: Advancing enteric disease treatments: probiotics and antimicrobial strategies

**DOI:** 10.3389/fimmu.2026.2023428

**Published:** 2026-10-28

**Authors:** Vijay Morampudi

**Affiliations:** Department of Biochemistry, School of Life Sciences, University of Hyderabad, Hyderabad, Telangana, India

**Keywords:** antimicrobial resistance, biofilms, enteric disease, gut microbiota, host–microbiome interactions, immune regulation, intestinal barrier, probiotics

## Background and scope

Enteric diseases arise from complex interactions among intestinal pathogens, the gut microbiome, the epithelial barrier, and host immune responses. Although antimicrobial therapy remains an important component of managing bacterial enteric infections, the increasing burden of antimicrobial resistance (AMR) has intensified the need for complementary therapeutic approaches (1). Antimicrobial exposure can also substantially alter the intestinal microbial ecosystem and its resistome (2). At the same time, biofilm formation and pathogen colonization can contribute to pathogen persistence and treatment challenges. Increasing recognition of the gut microbiome as an active regulator of intestinal homeostasis and immunity has therefore created opportunities to develop interventions that influence pathogens while preserving or restoring microbial and host functions (3, 4).

This Research Topic was established to examine how probiotics and other antimicrobial or microbiome-directed strategies could contribute to the prevention and management of enteric disease in the setting of increasing antimicrobial resistance. Particular emphasis was placed on interactions among the gut microbiome, intestinal pathogens, biofilm formation, epithelial integrity, and host immune responses. The eight contributions assembled in this Research Topic address these questions from complementary perspectives, spanning direct pathogen control, microbiota and metabolic remodeling, barrier protection, immune regulation, and emerging approaches to targeted microbial intervention.

## Pathogen control and colonization

Several contributions examined approaches that directly influence enteric pathogens and their ability to establish infection. Vasamsetti et al. demonstrated that *Lactobacillus johnsonii* inhibited *Enteropathogenic Escherichia coli* growth, disrupted pre-formed biofilms, and displaced adherent pathogens *in vitro*. In an antibiotic-perturbed *Citrobacter rodentium* mouse model, oral administration reduced pathogen loads by 3–4 log units, alleviated epithelial ulceration and inflammatory infiltration, and preserved epithelial architecture. Mechanistic analyses identified nutrient competition and secreted low-molecular-weight antimicrobial activity, while metabolomic profiling provided candidate molecules for future targeted validation. (Vasamsetti et al.)

El-Kafrawy et al. reviewed antibody-based functional silencing of gut pathobionts. Rather than assuming that therapeutic success necessarily requires eradication, the review examines mucosal antibodies, monoclonal antibodies and Immunoglobulin Y (IgY) as approaches that may attenuate virulence while preserving microbial community architecture. Importantly, the authors also identify evolutionary and ecological conditions under which retained pathobionts could re-emerge, emphasizing that functional silencing and eradication may represent context-dependent complementary strategies. (El-Kafrawy et al.).

Together, these studies illustrate approaches to pathogen control that include biofilm disruption, competitive exclusion, antimicrobial activity, and selective attenuation of pathogenic functions.

## Microbiota remodeling and microbial functions

A second theme is the ability of probiotic interventions to influence the intestinal microbial ecosystem and its metabolic functions. Feng et al. showed that *Enterococcus faecium* HDRsEf1 administration in experimental models was associated with changes in gut microbial composition and metabolites, together with reduced inflammatory and oxidative-stress markers and increased interleukin-10 (IL-10) and superoxide dismutase (SOD). The study also reported changes in microbial genera and enrichment of metabolites associated with beneficial host responses. (Feng et al.)

Peng et al. demonstrated that *Lactobacillus plantarum* ZG-7 alleviated intestinal injury caused by avian pathogenic *Escherichia coli* (APEC) infection in Muscovy ducks. Probiotic treatment improved growth performance, mitigated epithelial injury, and altered the intestinal microbial community, including enrichment of *Bacteroides*. The authors concluded that ZG-7 helped maintain beneficial microbial composition and protect epithelial barrier function during APEC infection. (Peng et al.)

The review by Zhuo et al. extends this concept to intestinal dysfunction associated with weaning stress in piglets. It emphasizes probiotic-mediated remodeling of the microbial ecosystem and restoration of microbial metabolic pathways involving short-chain fatty acids, secondary bile acids, and tryptophan metabolism, linking these changes with intestinal barrier repair, immune homeostasis and redox balance. (Zhuo et al.)

These findings suggest that probiotic activity may involve coordinated changes in microbial composition, microbial metabolism, and host physiology rather than direct pathogen inhibition alone.

## Barrier integrity, immune regulation and broader microbiome effects

The Topic also highlights interactions between microbial interventions, intestinal barrier integrity and host inflammatory responses. The *L. plantarum* study demonstrated mitigation of APEC-associated epithelial injury and protection of intestinal barrier function, while the *E. faecium* study associated probiotic administration with altered inflammatory and antioxidant responses. (Peng et al.; Feng et al.)

Prete et al. provided preliminary ex vivo evidence that fermentation of omega-3 polyunsaturated fatty acids (ω-3 PUFAs) with *Bacillus megaterium* produced a formulation that reduced selected pro-inflammatory signals and promoted a macrophage profile associated with resolution of inflammation in human ulcerative-colitis and healthy colon biopsies. The findings provide a basis for further *in vivo* and clinical investigation of this synbiotic approach. (Prete et al.)

The reviews by Xu et al. and Lin et al. broaden the biological context of microbiome-directed interventions. Xu et al. examine relationships between gut microbiota, iron metabolism, vitamin synthesis and immune regulation in anemia, whereas Lin et al. review links between the gut microbiota, immune-inflammatory responses, metabolism, intestinal barrier function and bone health, including probiotic, prebiotic and dietary interventions. (Xu et al.; Lin et al.) Although these reviews extend beyond enteric infection, they reinforce the wider significance of the gut microbiome as a mediator of host physiology and immune regulation.

## Broader significance and future directions

Taken together, the eight contributions highlight the interconnected roles of pathogens, the microbial ecosystem, the intestinal barrier, and host immune responses in enteric disease. At the same time, the findings remain strain- and context-dependent, with most experimental evidence derived from *in vitro*, animal or ex vivo models. Future work should establish reproducible strain-specific mechanisms, standardized formulations and outcome measures, while systematically addressing safety, AMR implications and translation to well-designed human studies.

## Conclusion

This Research Topic brings together complementary evidence for pathogen-directed and microbiome-directed approaches to enteric disease. Its contributions range from biofilm disruption and pathogen exclusion to microbiota remodeling, metabolic regulation, barrier protection and immune modulation. Together, these studies support the development of more targeted interventions that address enteric pathogens while considering the microbial ecosystem and host environment in which disease occurs. Further mechanistic and clinical studies will determine how these approaches can best complement existing antimicrobial therapies.
